# Supplementary material for: Increased interregional virus exchange and nucleotide diversity outline the expansion of chikungunya virus in Brazil
Source: Nat Commun. 2023 Jul 21;14:4413. doi: 10.1038/s41467-023-40099-y (PMC10362057; doi:10.1038/s41467-023-40099-y)
Supplement: Supplementary file 1 — Supplementary Information [file 41467_2023_40099_MOESM1_ESM.pdf]

Increased interregional virus exchange and nucleotide diversity outline the expansion of the chikungunya virus ECSA lineage in Brazil

Supplementary Information

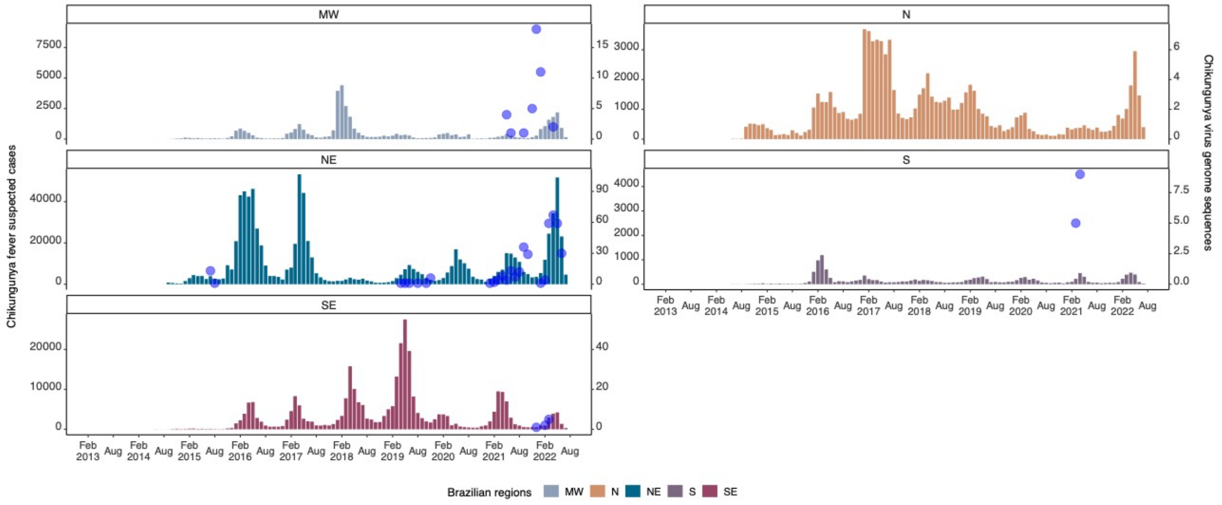

Supplementary Fig. 1. Number of chikungunya virus genomic sequences by number of cases. Bar plot of the number of notified suspected cases of chikungunya fever reported in different Brazilian geographic regions. The Brazilian chikungunya virus genomes generated in this study are indicated by light blue circles over the bars. Bar colors indicate different geographic regions in Brazil, indicated by the legend.

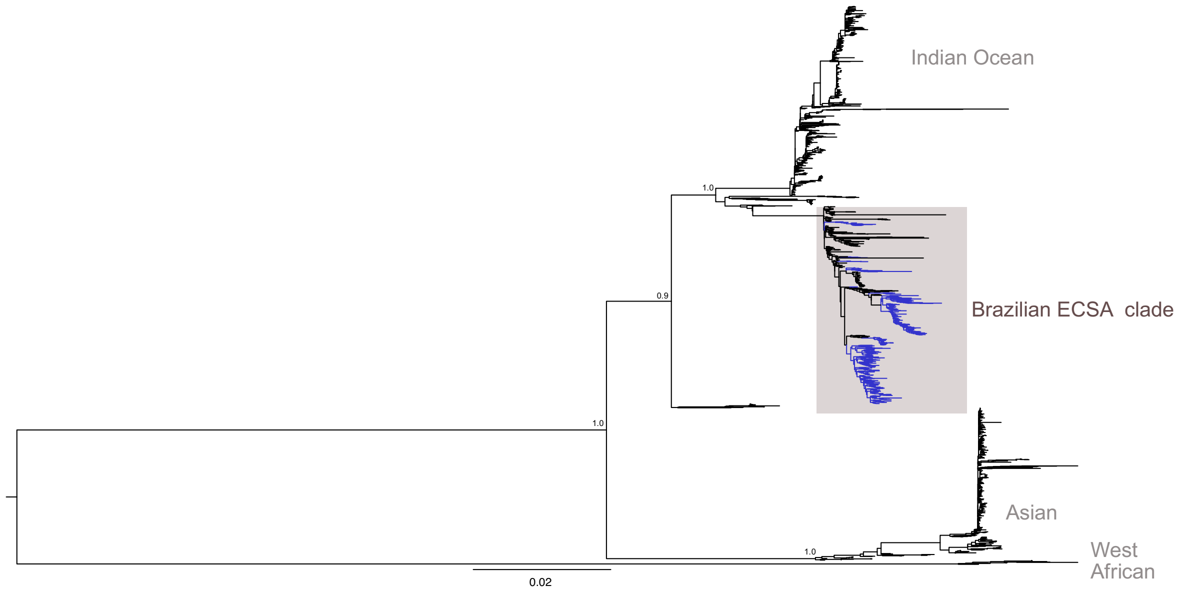

Supplementary Fig. 2. Global phylogeny of CHIKV lineages. Maximum Likelihood (ML) phylogeny was reconstructed using 1,987 CHIVK sequences, including the new genomes and others available on GenBank (see methods in the main text). New Brazilian genomes are indicated by blue branches in the Brazilian ECSA clade. Numbers are clade ultrafast bootstrap support values.

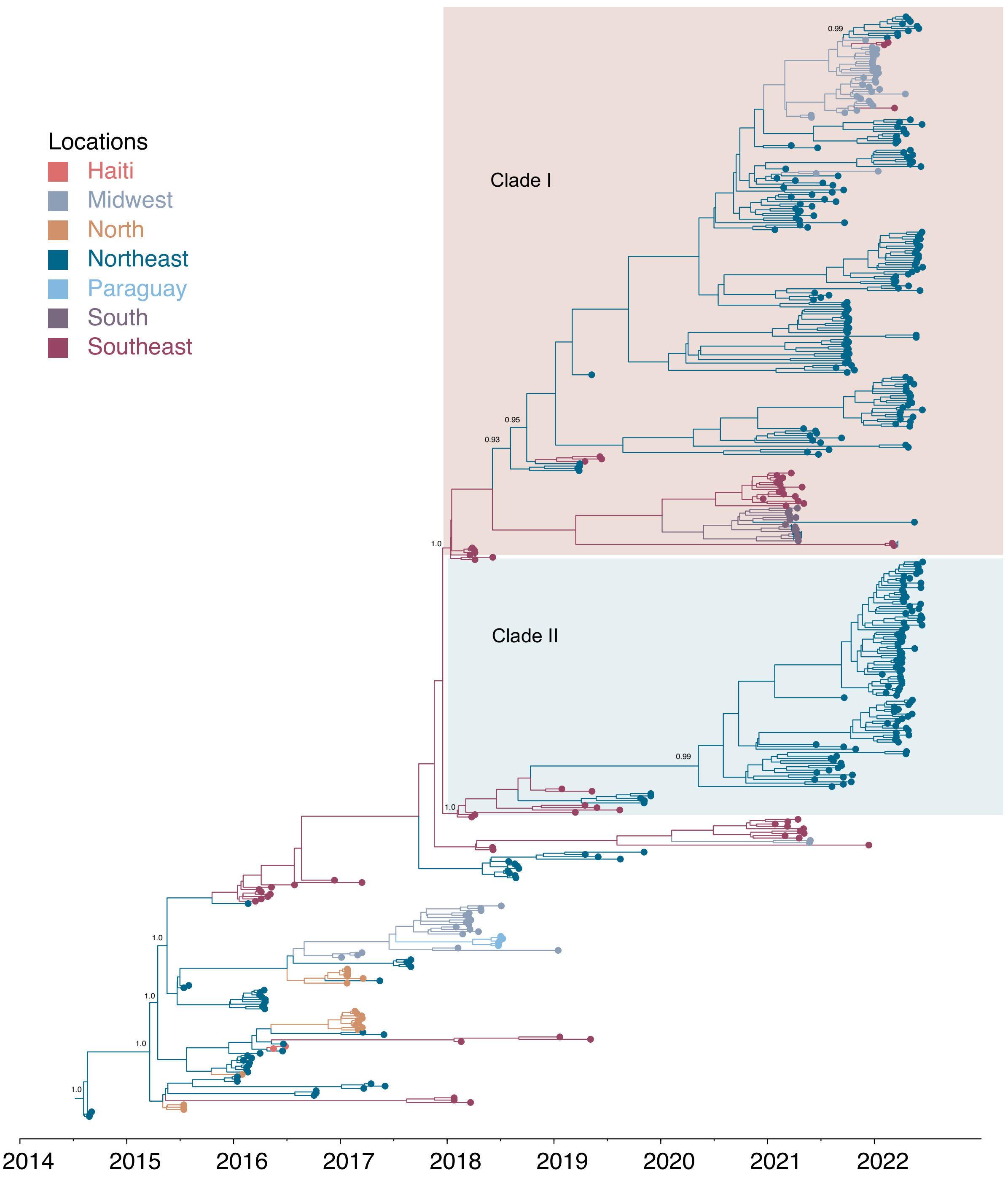

Supplementary Fig. 3. **Ancestral locations of viruses from the CHIKV ECSA lineage circulating in Brazil**. Maximum Clade Credibility tree reconstructed under a Bayesian phylogeography in discrete space using sequences (total n=471) from Brazil, Paraguay and Haiti (see methods in the main text)**.** Location probabilities of clades I and II as well as of main early branches estimated to be originated in the Northeast of Brazil are represented by numbers placed near nodes. Location probabilities of other clades were omitted for clarity. Branch colors represent the estimated ancestral location.

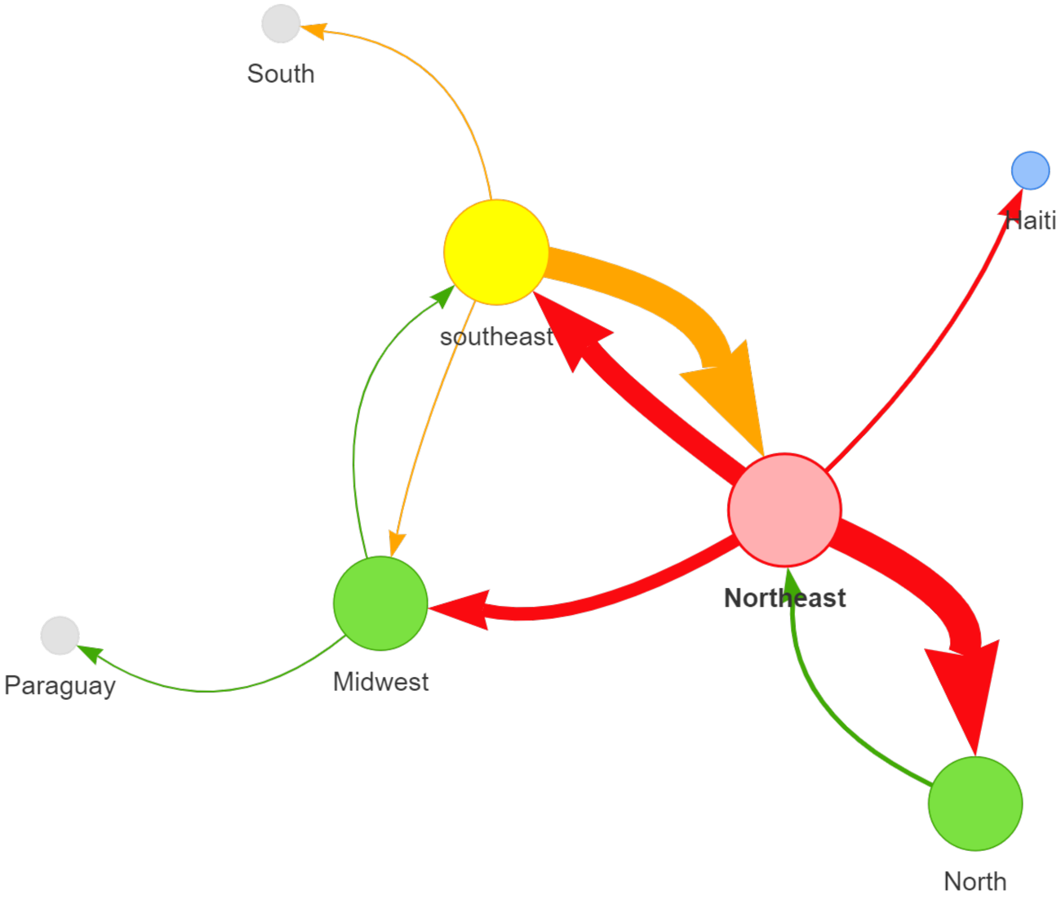

Supplementary Fig. 4. **Transmission network of CHIKV in Brazil**. The network was inferred from the transitions between geographic states from the Bayesian phylogeography under a discrete space model. Arrows indicate directionality and their thickness indicates transition frequencies. Node size was scaled by source hub ratio values estimated for each location using StrainHub tool v1.1.2. Colors are arbitrary.

Supplementary Table 1. Centrality Metrics employed to measure transmission networks generated by the StrainHub tool.

Metastates	Degree Centrality	Indegree Centrality	Outdegree Centrality	Betweenness Centrality	Closeness Centrality	Source Hub Ratio
Paraguay	1	1	0	0	0.07	0.00
Southeast	5	2	3	6	0.11	0.60
Northeast	6	2	4	9	0.13	0.67
Haiti	1	1	0	0	0.08	0.00
Midwest	4	2	2	3	0.11	0.50
North	2	1	1	0	0.08	0.50
South	1	1	0	0	0.07	0.00

Supplementary Table 2. Detailed site-by-site results from the BUSTED analysis for the envelope gene of clade I and II.

Codon	ER (ω>1)[1]	LogL	Epost[α][2]	LR[3]
Clade I				
187	187,712.29	-78.412	0.294	12.721
372	742.301	-31.012	1.07	6.651
699	344.611	-92.661	0.294	3.369
60	211.964	-79.268	0.294	2.566
62	57.168	-17.817	3.408	4.991
Clade II				
187	213,975.17	-78.617	36.082	10.86
186	92,991.85	-62.607	36.082	7.669
371	726.104	-17.444	2.287	6.078
121	499.333	-15.376	0.6	7.502

184	105.634	-59.121	35.724	0.507
29	96.931	-36.048	33.526	4.941
60	19.358	-78.757	36.082	1.279
370	12.692	-42.632	36.074	1.966

[1]Evidence ratio for positive selection.
[2]Posterior mean of the synonymous rate, α.
[3]Site log-likelihood ratio contribution.

Supplementary Table 3. Sites under positive diversifying selection identified by HYPHY-MEME in the envelope gene of the clades I and II.

Site	alpha	beta+	LRT	p-value
Clade I				
33	0	5.58	4.31	0.05
60	3.62	364.35	7.88	0.01
62	0	284.76	8.45	0.01
138	0	5.28	4.11	0.06
184	0	58.06	7.23	0.01
187	1.09	1521.76	21.03	0
282	0	2.31	3.43	0.09
372	0	6084.51	18.38	0
441	0	5.35	6.41	0.02
539	0	9.23	4.23	0.06
695	0	556.34	9.8	0
699	0	131.88	11.93	0
Clade II				
29	0	315.97	9.55	0
53	0	3.7	3.71	0.07
60	4.18	469.33	4.83	0.04
121	0	181.66	8.77	0.01

138	0	4.53	3.31	0.09
184	0	133.16	8.43	0.01
186	4.7	988.28	12.58	0
187	2.1	1124.28	20.44	0
371	0	256.38	9.31	0
432	0	4.87	3.88	0.07
441	0	3.16	3.38	0.09
539	0	70.36	4.82	0.04
699	0	9.05	3.67	0.08

alpha: synonymous substitution rate at a site.

beta+: non-synonymous substitution rate at a site for the positive selection category.

LRT: likelihood ratio test statistic for episodic diversification.

p-value: asymptotic p-values with a significance level alpha = 0.01.
