# Supplementary material for: Increased interregional virus exchange and nucleotide diversity outline the expansion of chikungunya virus in Brazil
Source: Nat Commun. 2023 Jul 21;14:4413. doi: 10.1038/s41467-023-40099-y (PMC10362057; doi:10.1038/s41467-023-40099-y)
Supplement: Supplementary file 3 — Description of Additional Supplementary Files [file 41467_2023_40099_MOESM3_ESM.pdf]

Description of Additional Supplementary Files

File Name: Supplementary Data 1
Description: Sample information, sequencing statistics and PCR primers sequences.

File Name: Supplementary Data 2
Description: Maximum Likelihood tree file reconstructed using the global dataset.
