# Supplementary material for: Increased interregional virus exchange and nucleotide diversity outline the expansion of chikungunya virus in Brazil
Source: Nat Commun. 2023 Jul 21;14:4413. doi: 10.1038/s41467-023-40099-y (PMC10362057; doi:10.1038/s41467-023-40099-y)
Supplement: Supplementary file 6 — Reporting Summary [file 41467_2023_40099_MOESM6_ESM.pdf]

nature portfolio

Corresponding author(s): Marta Giovanetti

Last updated by author(s): Jun 27, 2023

Reporting Summary

Nature Portfolio wishes to improve the reproducibility of the work that we publish. This form provides structure for consistency and transparency in reporting. For further information on Nature Portfolio policies, see our Editorial Policies and the Editorial Policy Checklist.

Statistics

For all statistical analyses, confirm that the following items are present in the figure legend, table legend, main text, or Methods section.

n/a	Confirmed	
☐	☒	The exact sample size (*n*) for each experimental group/condition, given as a discrete number and unit of measurement
☐	☒	A statement on whether measurements were taken from distinct samples or whether the same sample was measured repeatedly
☐	☒	The statistical test(s) used AND whether they are one- or two-sided<br>*Only common tests should be described solely by name; describe more complex techniques in the Methods section.*
☒	☐	A description of all covariates tested
☒	☐	A description of any assumptions or corrections, such as tests of normality and adjustment for multiple comparisons
☐	☒	A full description of the statistical parameters including central tendency (e.g. means) or other basic estimates (e.g. regression coefficient) AND variation (e.g. standard deviation) or associated estimates of uncertainty (e.g. confidence intervals)
☐	☒	For null hypothesis testing, the test statistic (e.g. *F*, *t*, *r*) with confidence intervals, effect sizes, degrees of freedom and *P* value noted<br>*Give P values as exact values whenever suitable.*
☐	☒	For Bayesian analysis, information on the choice of priors and Markov chain Monte Carlo settings
☒	☐	For hierarchical and complex designs, identification of the appropriate level for tests and full reporting of outcomes
☒	☐	Estimates of effect sizes (e.g. Cohen's *d*, Pearson's *r*), indicating how they were calculated

Our web collection on statistics for biologists contains articles on many of the points above.

Software and code

Policy information about availability of computer code

Data collection	All information, when relevant, can be found in the Methods section of the manuscript.
Data analysis	Guppy v6.0.1 was used for basecalling. MAFFT v.7. was used for sequence alignment. IQ-TREE 2.1.1 software was used for reconstruction of Maximum likelihood phylogenies. TempEst v.1.5.167 was used to assess presence of temporal signal in sequence datasets. BEAST v1.10.4 was used to perform Bayesian analysis of molecular sequences using MCMC to infer time-measured phylogenies. TreeAnnotator v1.10.4 was used to summarize trees. Tracer v1.7.2 was used visualize statistics from Bayesian analysis. SPREAD 4 was used to visualize state transitions inferred in the Bayesian phylogeographic approach. StrainHub online tool v1.1.2 was used to infer a transmission network. MALVIRUS v1.0.2 was used to extract single nucleotide variants from aligned genome sequences. HYPHY 2.5.42 software was used to assess selection pressure on genomic sequences. Box plots and bar charts were created using Rstudio 2022.12.0.

For manuscripts utilizing custom algorithms or software that are central to the research but not yet described in published literature, software must be made available to editors and reviewers. We strongly encourage code deposition in a community repository (e.g. GitHub). See the Nature Portfolio guidelines for submitting code & software for further information.

Data

Policy information about availability of data

All manuscripts must include a data availability statement. This statement should provide the following information, where applicable:

- Accession codes, unique identifiers, or web links for publicly available datasets
- A description of any restrictions on data availability
- For clinical datasets or third party data, please ensure that the statement adheres to our policy

New sequences have been deposited in GenBank under accession numbers OQ759652-OQ760076 listed in Supplementary Data 1. Input files used for the phylogenetic and mutational profile analyses are available at https://doi.org/10.6084/m9.figshare.22335331.v2.

Research involving human participants, their data, or biological material

Policy information about studies with human participants or human data. See also policy information about sex, gender (identity/presentation), and sexual orientation and race, ethnicity and racism.

Reporting on sex and gender	Patients sex data (female or male) were collected from self-report forms filled by patients upon sample collection at local public laboratories. Research team was not able to access patients for consent collection, and such limitation was described in the study protocol reviewed and approved by Research Ethics Committee. 246 participants were reported as female, while 179 were reported as male. Descriptive statistics (mean, median, standard deviation) and Mann-Whitney U test on median years of age with a significance level alpha = 0.05 were performed using participants sex data.
Reporting on race, ethnicity, or other socially relevant groupings	Socially constructed variables were not reported in this study since this information was not available.
Population characteristics	Patients' mean age upon sample collection was similar for both females and males (famale= mean 39.10 SD± 18.83; male= mean 39.37 SD± 22.03), with 57.88% (n=246) of the participants identified as female. The clinical status of patients at the time of sample collection, and travel history data were not available for these samples.
Recruitment	There was no specific criteria for patient recruitment. Samples were obtained from patients that actively sought medical care from local clinics after presenting symptoms such as fever and myalgia.
Ethics oversight	Study protocol was reviewed and approved by Research Ethics Committee of the Universidade Federal de Minas Gerais with approval No. 32912820.6.1001.5149.

Note that full information on the approval of the study protocol must also be provided in the manuscript.

Field-specific reporting

Please select the one below that is the best fit for your research. If you are not sure, read the appropriate sections before making your selection.

☒ Life sciences ☐ Behavioural & social sciences ☐ Ecological, evolutionary & environmental sciences

For a reference copy of the document with all sections, see nature.com/documents/nr-reporting-summary-flat.pdf

Life sciences study design

All studies must disclose on these points even when the disclosure is negative.

Sample size	Nanopore sequencing was performed on 425 selected CHIKV-positive samples provided by Brazilian Central Public Health Laboratories (LACEN) from 12 states across 4 geographic regions (Northeast, Midwest, Southeast, and South) of Brazil during the years 2021-2022. No sample size calculation was performed because these samples are residual samples (serum or plasma) available during an epidemic and they were obtained from the epidemiological surveillance routine from local Laboratories (LACEN). This sample size is sufficient for this study as it corresponds to more than twice the amount of sequences available at NCBI from Brazil. Sample size-related study limitations that might apply have already been addressed in the manuscript.
Data exclusions	CHIKV RT-qPCR positive samples were selected for sequencing based on the cycle threshold value equal or greater that 30. Samples with Ct value less than 30 were not processed. Sequences with viral genome coverage less than 60% were excluded from the phylogenetic analyses and previously discussed by Thézé et al. 2018 (full citation in the manuscript). Samples 736.22_RED, FS0116, and FS0132 were discarded due to low genome coverage.
Replication	Bayesian analysis of molecular sequences using MCMC was performed in duplicate, with each run of 200 million states. The convergence of MCMC chains was checked using Tracer software.
Randomization	Access to participants was not possible since positive samples were obtained from the epidemiological surveillance routine of local public Laboratories. Allocation of participants/samples to experimental groups is not relevant to this study since this work aimed to characterize viral isolates from all positive samples with Ct value equal or greater than 30 and genome coverage greater than 60%.

Blinding	Blinding was not relevant to this study since there was no allocation of participants/samples to experimental groups.

Reporting for specific materials, systems and methods

We require information from authors about some types of materials, experimental systems and methods used in many studies. Here, indicate whether each material, system or method listed is relevant to your study. If you are not sure if a list item applies to your research, read the appropriate section before selecting a response.

Materials & experimental systems

n/a	Involved in the study
☒	☐ Antibodies
☒	☐ Eukaryotic cell lines
☒	☐ Palaeontology and archaeology
☒	☐ Animals and other organisms
☒	☐ Clinical data
☒	☐ Dual use research of concern
☒	☐ Plants

Methods

n/a	Involved in the study
☒	☐ ChIP-seq
☒	☐ Flow cytometry
☒	☐ MRI-based neuroimaging
